# Preoperative Lactate Dehydrogenase-to-Albumin Ratio as a Tumor–Host Biomarker of Early Recurrence and Survival in Resected Pulmonary Neuroendocrine Carcinomas: A Multicenter Observational Cohort Study

**DOI:** 10.3390/medicina62050946

**Published:** 2026-05-13

**Authors:** Hacer Boztepe Yesilcay, Asim Armagan Aydin, Ahmet Baklaci, Abdurrahman Aykut, Ahmet Unlu, Merve Turan, Ismail Oguz Kara, Ramazan Oguz Yuceer, Muhammed Fatih Sagiroglu, Sencan Akdag, Mustafa Yildiz

**Affiliations:** 1 Department of Thoracic Surgery, Antalya Training and Research Hospital, University of Health Sciences, 07100 Antalya, Turkey; sencanakdag@hotmail.com; 2 Department of Clinical Oncology, Antalya Training and Research Hospital, University of Health Sciences, 07100 Antalya, Turkey; drarmaganaydin@gmail.com (A.A.A.); md.ahmetunlu@gmail.com (A.U.); drmyildiz@yahoo.com (M.Y.); 3 Department of Medical Oncology, Faculty of Medicine, Aydin Adnan Menderes University, 09100 Aydin, Turkey; baklaci.ahmet@gmail.com (A.B.); drmerveturan@gmail.com (M.T.); 4 Department of Medical Oncology, Faculty of Medicine, Çukurova University, 01330 Adana, Turkey; dr.abdurrahmanaykut@gmail.com (A.A.); iokara@cu.edu.tr (I.O.K.); 5 Department of Pathology, Faculty of Medicine, Sivas Cumhuriyet University, 58140 Sivas, Turkey; r.yuceer66@hotmail.com; 6 Department of Medical Oncology, Antalya City Hospital, 07070 Antalya, Turkey; dr.mfsagiroglu@gmail.com

**Keywords:** pulmonary neuroendocrine carcinoma, small cell lung cancer, large cell neuroendocrine carcinoma, lactate dehydrogenase, albumin, lactate dehydrogenase-to-albumin ratio, recurrence, survival, immune inflammation, nutrition and cancer

## Abstract

*Background and Objectives*: Pulmonary neuroendocrine carcinomas (NECs) are characterized by aggressive clinical behavior and heterogeneous postoperative outcomes. Early recurrence, often reflecting occult micrometastatic disease, remains a key determinant of prognosis and is insufficiently captured by conventional staging systems. We hypothesized that the lactate dehydrogenase-to-albumin ratio (LAR), as an integrative tumor–host biomarker, may provide biologically informed risk stratification in this setting. *Materials and Methods*: We conducted a multicenter retrospective cohort study including 88 patients with resected small cell lung cancer (SCLC) or large cell neuroendocrine carcinoma (LCNEC). Preoperative LAR and comparator inflammatory indices were evaluated. The primary endpoints were disease-free survival (DFS) and overall survival (OS), with early recurrence (≤12 months) as a prespecified secondary endpoint. Time-dependent receiver operating characteristic analyses, Cox proportional hazards models, and logistic regression analyses were applied within a predefined analytical framework. *Results*: Using a cut-off derived from 12-month DFS (LAR = 45.58), elevated LAR was associated with significantly shorter DFS (median 12.3 vs. 26.1 months; *p* = 0.018) and OS (median 20.7 vs. 52.8 months; *p* = 0.010). In multivariable analyses, LAR remained independently associated with both DFS (HR 1.012, 95% CI 1.001–1.023; *p* = 0.037) and OS (HR 1.016, 95% CI 1.005–1.027; *p* = 0.003). Elevated LAR was also associated with an increased risk of early recurrence (adjusted OR 4.656, 95% CI 1.520–14.262; *p* = 0.007). In time-dependent receiver operating characteristic (ROC) analyses, LAR demonstrated the highest overall discriminatory performance across evaluated biomarkers and showed a statistically significant advantage over neutrophil-to-lymphocyte ratio (NLR) for 24-month OS. *Conclusions*: Preoperative LAR captures a clinically relevant tumor–host phenotype associated with early disease progression and adverse survival outcomes in resected pulmonary NECs. As a biologically integrative and readily accessible biomarker, LAR may complement existing risk stratification strategies in this heterogeneous disease context. Prospective validation and integration into multimodal risk models are warranted.

## 1. Introduction

Pulmonary neuroendocrine carcinomas (NECs), including small cell lung cancer (SCLC) and large cell neuroendocrine carcinoma (LCNEC), represent a highly aggressive spectrum of thoracic malignancies characterized by rapid proliferation, early systemic dissemination, and poor clinical outcomes [1,2]. Pulmonary NECs account for a clinically significant subset of lung cancers, largely driven by the high prevalence of SCLC, which constitutes approximately 15% of all lung cancer cases, whereas LCNEC represents a rarer but biologically similar entity. Tobacco exposure remains the predominant risk factor, particularly for SCLC, which is strongly associated with cumulative smoking burden, while the etiologic determinants of LCNEC are less clearly defined but appear to share similar smoking-related associations. Even in patients with apparently localized disease undergoing curative-intent surgical resection [3], recurrence rates remain substantial, and survival outcomes are markedly heterogeneous, underscoring the limited capacity of conventional clinicopathologic staging to fully capture the underlying biological complexity of these tumors [4,5]. A defining clinical challenge in this setting is the occurrence of early postoperative recurrence, frequently within the first year after surgery, which likely reflects the presence of occult micrometastatic disease at diagnosis [6,7,8]. This early relapse phenotype highlights a critical gap in current risk stratification strategies, as patients with biologically aggressive disease remain indistinguishable from those with more indolent trajectories using standard staging parameters alone [9]. In this context, there is an urgent need for clinically accessible, preoperative biomarkers capable of capturing the integrated tumor–host dynamics that underpin early disease progression and adverse survival outcomes in resectable pulmonary NECs.

The biological rationale for the lactate dehydrogenase-to-albumin ratio (LAR) as an integrative biomarker is based on its ability to capture complementary dimensions of tumor metabolism and host systemic physiology. Lactate dehydrogenase (LDH) reflects enhanced glycolytic flux and hypoxia-driven metabolic reprogramming, which are hallmarks of aggressive tumor biology associated with rapid proliferation and metabolic adaptation [10,11]. At a biochemical level, LDH catalyzes the interconversion of pyruvate to lactate, a key step in anaerobic glycolysis that enables continued ATP production under hypoxic conditions. The accumulation of lactate, beyond its established role as a biomarker of tissue hypoperfusion, contributes to an immunosuppressive tumor microenvironment and facilitates metabolic adaptation in cancer [12]. Beyond serving as a surrogate for tumor burden, elevated LDH is closely linked to lactate accumulation within the tumor microenvironment, which promotes immune evasion through the suppression of cytotoxic T-cell activity, polarization of tumor-associated macrophages, and inhibition of dendritic cell function [10,13,14]. Serum albumin is a downstream marker of cancer-associated systemic inflammation, reflecting cytokine-mediated hepatic reprioritization, increased protein catabolism, and impaired nutritional and physiological reserves [15,16]. Hypoalbuminemia is associated with a proinflammatory state driven by interleukin-6 and related mediators, contributing to host vulnerability and facilitating tumor progression [17]. By integrating these biologically interconnected processes, the LAR provides a composite measure of tumor-driven metabolic stress and host resilience. Rather than reflecting isolated pathways, it captures a dynamic tumor–host ecosystem in which metabolic activation and systemic inflammation are tightly coupled and biologically reinforced, offering a mechanistically grounded perspective on disease biology in aggressive malignancies such as pulmonary NECs.

A growing body of literature has explored blood-based biomarkers that reflect systemic inflammation and nutritional status across a range of malignancies, including pulmonary NECs. In this context, indices such as the neutrophil-to-lymphocyte ratio (NLR) and systemic immune-inflammation index (SII) have been investigated and shown to be associated with adverse clinical outcomes, underscoring the relevance of host inflammatory responses in shaping disease behavior [18,19,20,21]. In parallel, composite indices incorporating nutritional and immunological parameters, such as the prognostic nutritional index (PNI), have also demonstrated prognostic significance in resected high-grade pulmonary neuroendocrine carcinomas, highlighting the contribution of host physiological reserves to postoperative outcomes [22]. Taken together, these observations highlight the prognostic relevance of host-related biomarkers in pulmonary NECs; however, they have primarily focused on the individual components of tumor–host interactions, particularly systemic inflammation or nutritional status. Given the biological complexity of these tumors, characterized by pronounced metabolic reprogramming alongside systemic host responses, an integrative approach that captures both tumor-driven metabolic activity and host conditions may offer a more comprehensive understanding of disease behavior. This consideration is particularly relevant in the setting of resectable disease, where early recurrence represents a key clinical challenge and may reflect underlying tumor–host dynamics that are not fully captured by conventional biomarkers.

In this context, we conducted a multicenter, real-world cohort study to evaluate the clinical and biological relevance of preoperative blood-based biomarkers in patients with resected pulmonary NECs. Given the aggressive clinical course and marked heterogeneity observed even in operable early-stage disease, the study was specifically designed to examine early postoperative recurrence as a clinically distinct and biologically aggressive phenotype. In contrast to prior studies that have primarily evaluated inflammation-based indices or individual components of the tumor–host interaction, we focused on the LAR as an integrative biomarker reflecting both tumor metabolic activity and host systemic condition. Within a predefined analytical framework, we assessed its association with early recurrence and survival outcomes and compared its discriminative performance with established inflammatory indices. We hypothesized that LAR may provide incremental prognostic insight beyond conventional markers by capturing the interplay between tumor metabolism and host physiology.

## 2. Materials and Methods

### 2.1. Study Design and Patient Selection

This multicenter, real-world, retrospective cohort study was conducted across three tertiary oncology centers in Turkey: the Department of Medical Oncology at Çukurova University Faculty of Medicine, the Department of Medical Oncology at Aydın Adnan Menderes University Faculty of Medicine, and the Department of Medical Oncology at the University of Health Sciences Antalya Training and Research Hospital. This study aimed to evaluate the clinical relevance of preoperative blood-based biomarkers, with a particular focus on LAR, in relation to clinical outcomes and recurrence patterns following curative-intent surgical resection. We retrospectively screened consecutive adult patients with histologically confirmed pulmonary NECs, including SCLC and LCNEC, who underwent surgical resection between January 2014 and November 2023.

**Figure 1:**
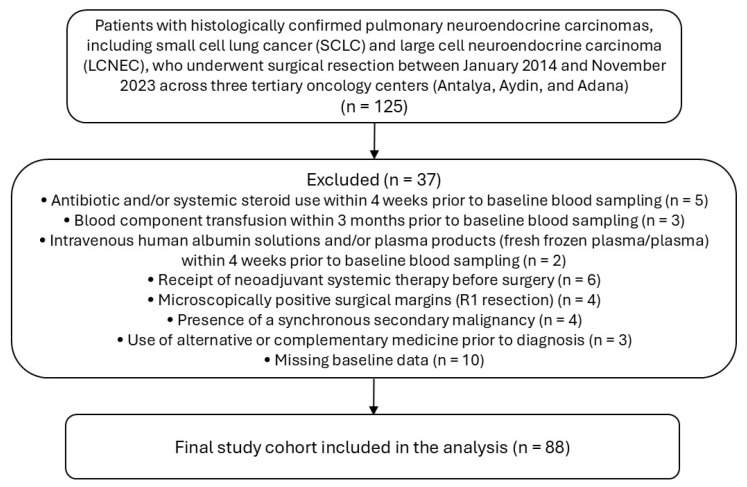
Study Cohort Flow Diagram (CONSORT Framework).

To minimize potential confounding, patients were excluded if they had evidence of active infection or had received antibiotics or systemic corticosteroids within 4 weeks prior to baseline blood sampling, blood transfusion within 3 months, intravenous albumin, or plasma products within 4 weeks. Additional exclusion criteria included prior malignancy requiring active treatment, receipt of neoadjuvant systemic therapy before surgery, use of alternative or complementary therapies prior to diagnosis, microscopically positive surgical margins (R1 resection), and incomplete baseline clinical or laboratory data. A total of 88 patients met the eligibility criteria and were included in the final analysis (Figure 1).

The primary endpoints were disease-free survival (DFS) and overall survival (OS), whereas early recurrence (defined as recurrence within 12 months after surgery) was prespecified as a clinically relevant secondary endpoint reflecting aggressive disease biology.

The study was conducted in accordance with the Declaration of Helsinki and approved by the Institutional Review Board of the University of Health Sciences Antalya Training and Research Hospital and the Antalya Provincial Health Directorate (approval no. 2026-66; decision no. 6/2; 26 March 2026). The requirement for informed consent was waived because of the retrospective design and use of anonymized data.

Given the retrospective design of the study, no formal a priori sample size calculation was performed. Instead, all consecutive eligible patients meeting the predefined inclusion criteria during the study period were included to reflect real-world clinical practice and minimize selection bias.

### 2.2. Data Collection

Clinical, demographic, surgical, and pathological data were retrospectively extracted from electronic medical records using a standardized data collection framework at the participating centers.

Baseline variables included age, sex, smoking status, Eastern Cooperative Oncology Group (ECOG) performance status, and comorbidity status. Tumor-related characteristics included tumor laterality, histologic subtype (SCLC vs. LCNEC), pathological tumor stage (pT), nodal status (pN), clinical stage at diagnosis, lymphovascular invasion, perineural invasion, and primary tumor diameter.

Surgical variables included the type of resection (segmentectomy or lobectomy vs. pneumonectomy) and surgical approach (video-assisted thoracoscopic surgery [VATS] vs. open thoracotomy). Data on postoperative outcomes included recurrence status, site and pattern of recurrence (locoregional vs. systemic), and the development of brain metastases during follow-up.

Preoperative laboratory parameters were obtained from routine blood tests performed within seven days before surgical resection. These included complete blood count and standard biochemical measurements, with particular emphasis on LDH and serum albumin levels. All laboratory analyses were conducted as part of routine clinical practice at each participating center using standardized automated analyzers in accordance with institutional quality control procedures. Given the retrospective design, laboratory values were based on single determinations recorded in the clinical database prior to any surgical or oncological intervention.

### 2.3. Biomarker Definitions

The primary biomarker of interest was the preoperative LAR, calculated as the serum lactate dehydrogenase (U/L) divided by the serum albumin (g/dL) [23]. LAR was selected as a biologically integrative marker reflecting the interplay between tumor metabolic activity and host systemic conditions.

To contextualize the performance of LAR, a panel of established inflammation- and nutrition-based indices was included as a comparator biomarker. These included the NLR [18], monocyte-to-lymphocyte ratio (MLR) [24], SII [20], systemic inflammation response index (SIRI) [25], pan-immune-inflammation value (PIV) [26], C-reactive protein-to-albumin ratio (CAR) [27], and GINI index [28]. These indices were selected based on prior evidence supporting their association with clinical outcomes across malignancies and their representation of distinct aspects of host inflammatory and nutritional responses.

All biomarkers were initially analyzed as continuous variables to maintain statistical information. For clinical interpretability, LAR was subsequently dichotomized using an optimal cutoff value derived from receiver operating characteristic (ROC) analysis at the prespecified 12-month DFS time point based on the Youden index. To minimize the risk of overfitting and maintain analytical consistency, this cutoff was applied uniformly across all subsequent analyses, including survival and early recurrence endpoints, without further optimization.

The same methodological framework was applied to the comparator biomarkers to enable a consistent and unbiased comparison of their discriminative performances. Accordingly, cut-off values for all indices were derived using an identical approach and evaluated across predefined clinical endpoints.

### 2.4. Treatment and Follow-Up

All patients underwent curative-intent surgical resection according to contemporary thoracic oncology guidelines. The extent of resection and surgical approach were determined by multidisciplinary tumor boards at each participating center based on tumor characteristics, patient performance status, and feasibility of the surgery.

Given the retrospective multicenter design, variations in surgical management, perioperative care, and adjuvant treatment strategies were anticipated and considered reflective of real-world clinical practice. Decisions regarding adjuvant therapy were made at the discretion of the treating physicians, in accordance with prevailing clinical guidelines.

Postoperative follow-up was conducted through serial clinical assessments and cross-sectional imaging, primarily using thoracic computed tomography, at regular intervals or upon clinical suspicion of disease recurrence. Additional imaging modalities were used when clinically indicated. Disease recurrence was defined based on radiologic findings consistent with tumor relapse, with histopathological confirmation obtained when clinically indicated.

DFS was defined as the time from surgical resection to the first occurrence of disease recurrence or death from any cause, whichever occurred first. OS was defined as the time from surgery to death from any cause. Patients without events were censored on the date of the last follow-up.

### 2.5. Statistical Analysis

All statistical analyses were performed using R software (version 4.4.1; R Foundation for Statistical Computing, Vienna, Austria) and IBM SPSS Statistics (version 27.0; IBM Corp., Armonk, NY, USA). Categorical variables were summarized as frequencies and percentages and compared using the χ^2^ test or Fisher’s exact test, as appropriate. Continuous variables are reported as medians with interquartile ranges (IQRs). All statistical tests were two-sided, and a *p*-value < 0.05 was considered statistically significant.

The discriminative performance of LAR and comparator biomarkers was evaluated using time-dependent ROC curve analysis with inverse probability of censoring weighting (IPCW), focusing on prespecified clinically relevant time points of 12-month DFS and 24-month OS. The area under the curve (AUC) was calculated for each biomarker, and pairwise comparisons of the AUCs were performed using the DeLong test.

For clinical interpretability, the optimal cutoff values for LAR and the comparator indices were determined using the Youden index at the prespecified 12-month DFS time point. To minimize overfitting and maintain analytical consistency, these cutoff values were applied uniformly across all subsequent analyses, including survival and early recurrence endpoints, without further optimization.

Survival outcomes were analyzed using the Kaplan–Meier method and compared using the log-rank test. Associations with DFS and OS were evaluated using univariable and multivariable Cox proportional hazard regression models. The multivariable models included prespecified clinically relevant covariates, including age, ECOG performance status, comorbidity status, histologic subtype, and pathological staging parameters. Model construction was guided by clinical relevance rather than automated variable selection to reduce the risk of overfitting the model.

The association between LAR and early recurrence (defined as recurrence within 12 months after surgery) was assessed using logistic regression. Multivariable logistic models were adjusted for key clinical covariates to account for potential confounding factors. To minimize the risk of overfitting, the number of covariates included in the multivariable models was restricted relative to the number of observed outcome events, and variable selection was guided by clinical relevance rather than automated data-driven procedures. Multicollinearity was assessed using variance inflation factors (VIFs), with values < 2 considered indicative of no relevant collinearity. Internal model stability was further explored using bootstrap resampling with 1000 repetitions.

Subgroup analyses were conducted according to histologic subtype (SCLC vs. LCNEC) and nodal status (pN0 vs. pN+). Interaction terms were tested to evaluate the consistency of the association between the LAR and clinical outcomes across the predefined subgroups.

All biomarkers were analyzed as continuous and dichotomized variables based on predefined cut-off values to balance statistical robustness with clinical interpretability.

## 3. Results

### 3.1. Baseline Characteristics

Finally, 88 patients with resected early-stage pulmonary neuroendocrine carcinoma were included in the analysis. Based on the predefined preoperative LAR cutoff of 45.58, 37 (42.0%) and 51 (58.0%) patients were classified as LAR-low and LAR-high, respectively (Table 1).

The median age of the overall cohort was 63.0 years (interquartile range [IQR], 58.0–68.2), with comparable distributions between the LAR-low and LAR-high groups (63.0 [IQR, 55.0–67.0] vs. 65.0 [IQR, 58.0–69.5], respectively). The majority of patients were male (83.0%), and the sex distribution was similar across the LAR strata. Most patients had a good performance status, with 92.0% classified as ECOG 0–1, without significant intergroup differences. Likewise, no meaningful imbalances were observed in smoking status, comorbidity burden, or tumor laterality between the LAR-low and LAR-high cohorts.

Regarding tumor characteristics, the distribution of histologic subtypes (SCLC vs. LCNEC), pathological T stage (T1–2 vs. T3–4), nodal status (N0 vs. N+), and clinical stage (stage I–III) was well balanced between the two groups. Similarly, no significant differences were identified in lymphovascular or perineural invasion status or in primary tumor diameter. Surgical variables, including the type of resection and surgical approach (video-assisted thoracoscopic surgery vs. open thoracotomy), were comparable across the LAR-defined subgroups.

Among patients who developed disease progression, neither the incidence of brain metastasis at progression nor the pattern of progression (locoregional vs. systemic) differed significantly between the LAR groups. Overall, the baseline clinicopathological characteristics were well distributed between the LAR-low and LAR-high cohorts, indicating the absence of major imbalances that could confound subsequent survival analyses.

**Table 1:** Baseline Characteristics of the Study Cohort According to Preoperative Lactate Dehydrogenase-to-albumin Ratio Group (*n* = 88).

Variable	Category	Overall, n = 88(%)	Lactate Dehydrogenase-to-Albumin Ratio (LAR)		*p*
			Low-LAR, *n*(%)	High-LAR, *n*(%)	
Age, years	Median (IQR)	63.0 (58.0–68.2)	63.0 (55.0–67.0)	65.0 (58.0–69.5)	0.191
Sex	Female	15 (17.0)	6 (16.2)	9 (17.6)	0.860
	Male	73 (83.0)	31 (83.8)	42 (82.4)	
ECOG performance status	0–1	81 (92.0)	34 (91.9)	47 (92.2)	1.000
	≥2	7 (8.0)	3 (8.1)	4 (7.8)	
Smoking status	Never	8 (9.1)	4 (10.8)	4 (7.8)	0.716
	Ever	80 (90.9)	33 (89.2)	47 (92.2)	
Comorbidity	Yes	63 (71.6)	25 (67.6)	38 (74.5)	0.476
	No	25 (28.4)	12 (32.4)	13 (25.5)	
Tumor laterality	Left	41 (46.6)	19 (51.4)	22 (43.1)	0.446
	Right	47 (53.4)	18 (48.6)	29 (56.9)	
Histologic subtype	SCLC	43 (48.9)	18 (48.6)	25 (49.0)	0.973
	LCNEC	45 (51.1)	19 (51.4)	26 (51.0)	
Type of resection	Segmentectomy/Lobectomy	79 (89.8)	33 (89.2)	46 (90.2)	1.000
	Pneumonectomy	9 (10.2)	4 (10.8)	5 (9.8)	
Type of surgery	VATS	24 (27.6)	11 (29.7)	13 (26.0)	0.700
	Open thoracotomy	63 (72.4)	26 (70.3)	37 (74.0)	
pT stage subgroup	T1–2	74 (84.1)	30 (81.1)	44 (86.3)	0.511
	T3–4	14 (15.9)	7 (18.9)	7 (13.7)	
pN stage subgroup	N0	53 (60.2)	24 (64.9)	29 (56.9)	0.449
	N+	35 (39.8)	13 (35.1)	22 (43.1)	
Clinical stage	I	37 (42.0)	18 (48.6)	19 (37.3)	0.564
	II	32 (36.4)	12 (32.4)	20 (39.2)	
	III	19 (21.6)	7 (18.9)	12 (23.5)	
Lymphovascular invasion	Yes	51 (58.0)	20 (54.1)	31 (60.8)	0.528
	No	37 (42.0)	17 (45.9)	20 (39.2)	
Perineural invasion	Yes	24 (27.3)	9 (24.3)	15 (29.4)	0.597
	No	64 (72.7)	28 (75.7)	36 (70.6)	
Primary tumor diameter, mm	Median (IQR)	25.0 (18.0–42.0)	21.0 (18.0–40.0)	30.0 (18.0–42.0)	0.482
Brain metastasis at progression *	Yes	20 (35.1)	8 (40.0)	12 (32.4)	0.568
	No	37 (64.9)	12 (60.0)	25 (67.6)	
Progression pattern *	Locoregional	19 (33.3)	9 (45.0)	10 (27.0)	0.170
	Systemic	38 (66.7)	11 (55.0)	27 (73.0)	

**Abbreviations**: ECOG, Eastern Cooperative Oncology Group; IQR, interquartile range; LAR, lactate dehydrogenase-to-albumin ratio; LCNEC, large cell neuroendocrine carcinoma; SCLC, small cell lung cancer; VATS, video-assisted thoracoscopic surgery. Data are presented as median (IQR) or *n* (%). *p* values were calculated using the Mann–Whitney U test for continuous variables and the chi-square test or Fisher’s exact test for categorical variables, as appropriate. * Progression-related variables were analyzed among patients who developed disease progression (*n* = 57).

### 3.2. Receiver Operating Characteristic Analysis

Time-dependent ROC analyses were performed to evaluate the discriminatory performance of preoperative systemic biomarkers for 12-month DFS and 24-month OS.

For 12-month DFS, LAR demonstrated the highest AUC among all evaluated biomarkers (AUC 0.603, 95% CI 0.468–0.729), with an optimal cut-off value of 45.58 derived using the Youden index. Although the overall discriminatory capacity for DFS was modest across biomarkers, LAR consistently ranked as the best-performing biomarker.

For 24-month OS, LAR again showed the highest discriminatory performance (AUC 0.679, 95% CI 0.544–0.806), indicating a modest yet consistent ability to stratify patients according to their survival outcomes. CAR and the GINI index demonstrated intermediate performance, whereas other inflammatory indices, including NLR, SII, and PIV, showed limited discrimination ability.

Importantly, DeLong comparisons revealed that the AUC of LAR for 24-month OS was significantly higher than that of NLR (*p* = 0.041), while the differences between LAR and the other biomarkers were not statistically significant. No significant differences were observed between the biomarkers for the 12-month DFS.

**Figure 2:**
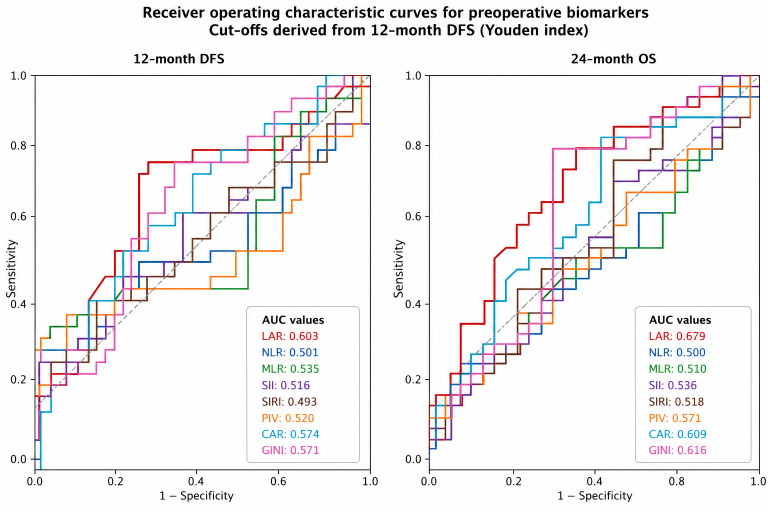
Receiver Operating Characteristic (ROC) Analysis of Systemic Inflammatory Biomarkers for Recurrence and Survival in Resected Pulmonary Neuroendocrine Carcinomas. Optimal biomarker cut-off values were derived from the 12-month DFS ROC curves using the Youden index and subsequently applied for both DFS and OS evaluations. The area under the curve (AUC) values for each biomarker are displayed in the lower right corner of each panel. Distinctly colored curves represent individual biomarkers, and the corresponding AUC values quantify their discriminatory performance for the specified clinical endpoints.

Taken together, these findings indicate that LAR provides the most consistent discriminatory performance across clinically relevant endpoints, despite the modest predictive capacity observed in this surgically treated early-stage cohort (Figure 2, Table 2).

### 3.3. Survival Analyses

**Figure 3:**
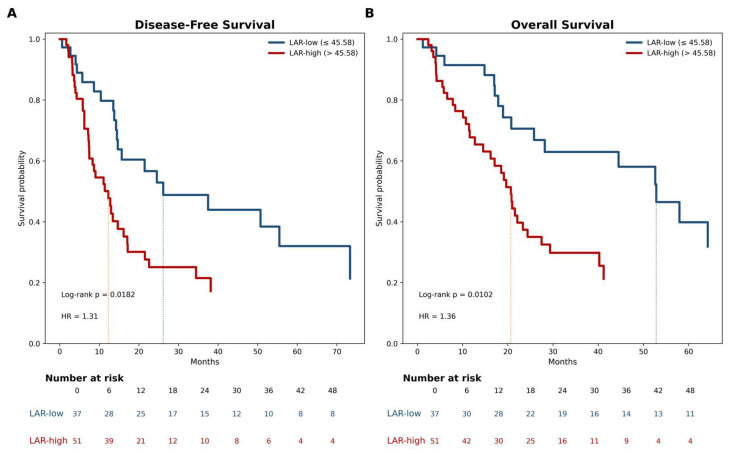
Kaplan–Meier Survival Analysis According to Preoperative Lactate Dehydrogenase-to-albumin Ratio in Resected Pulmonary Neuroendocrine Carcinomas. Optimal biomarker stratification was derived using the cut-off value identified from the 12-month disease-free survival receiver operating characteristic analysis (Youden index). Patients were categorized into LAR-low and LAR-high groups based on this threshold value. (**A**) Kaplan–Meier estimates of disease-free survival stratified by preoperative LAR status. (**B**) Kaplan–Meier estimates of overall survival stratified by preoperative LAR status. Survival differences between the groups were assessed using the log-rank test. The hazard ratios represent the relative risk associated with elevated LAR levels. The numbers below the curves indicate the number of patients at risk at each time point.

In the overall cohort, the median DFS and OS were 14.7 and 24.4 months, respectively. Kaplan–Meier analyses demonstrated a clear and clinically meaningful separation of the survival curves according to the preoperative LAR status. Using the cut-off derived from the 12-month DFS ROC analysis, patients in the LAR-high group experienced significantly shorter DFS than those in the LAR-low group (median DFS, 12.3 vs. 26.1 months; log-rank *p* = 0.018). A similar pattern was observed for OS, with markedly inferior survival in the LAR-high group (median OS, 20.7 vs. 52.8 months; log-rank *p* = 0.010). These differences translate into a substantial survival disadvantage associated with an elevated preoperative LAR, supporting its clinical relevance as a risk stratification marker. Overall, preoperative LAR effectively identified a subgroup of patients with a higher risk of early recurrence and mortality following curative-intent surgical resection (Figure 3).

### 3.4. Cox Regression Analyses

In the univariable Cox regression analyses, several clinicopathological factors were associated with survival outcomes. For DFS, pathologic nodal involvement (pN+) (HR 1.954, 95% CI 1.149–3.322, *p* = 0.013) was significantly associated with a shorter DFS. Among the evaluated biomarkers, LAR was the only marker significantly associated with DFS (HR 1.012, 95% CI 1.002–1.023, *p* = 0.024), whereas conventional inflammatory indices showed no significant prognostic value. For OS, LAR showed a strong and statistically significant association with an increased mortality risk (HR 1.017, 95% CI 1.007–1.027, *p* = 0.001). Comorbidity was also independently associated with worse OS (HR 2.054, 95% CI 1.025–4.117, *p* = 0.042), whereas ECOG performance status and nodal stage demonstrated borderline associations (Table 3).

In multivariable Cox regression analyses, LAR remained independently associated with both DFS and OS after adjusting for clinically relevant covariates. For DFS, LAR retained independent prognostic significance in Model 1 (HR 1.012, 95% CI 1.001–1.023, *p* = 0.037), and this association remained robust after additional adjustment for SIRI in Model 2 (HR 1.011, 95% CI 1.000–1.022, *p* = 0.044). Notably, SIRI was not independently associated with DFS (*p* = 0.272). For OS, LAR demonstrated consistent independent prognostic value across both models (Model 1: HR 1.016, 95% CI 1.005–1.027, *p* = 0.003; Model 2: HR 1.015, 95% CI 1.005–1.026, *p* = 0.005). In addition, ECOG performance status and comorbidities were independently associated with worse OS (Table 4).

Importantly, the prognostic effect of LAR remained stable across multiple models and was not attenuated after adjusting for established clinicopathological factors. Furthermore, the inclusion of SIRI did not materially alter the effect size of LAR, suggesting that LAR captures a biologically relevant tumor–host interaction not reflected by conventional inflammation-based indices (Table 3 and Table 4).

Collinearity diagnostics did not indicate relevant multicollinearity among the variables included in the multivariable models, with all VIF values below 2. In 1000-repetition bootstrap resampling, the association between LAR and OS remained stable across both multivariable models, with bootstrap-derived 95% confidence intervals of 1.006–1.029 for Model 1 and 1.006–1.031 for Model 2. For DFS, the direction of effect remained consistent, although the bootstrap-derived intervals were wider and crossed unity, with 95% confidence intervals of 0.999–1.022 for Model 1 and 0.997–1.022 for Model 2.

### 3.5. Early Recurrence Analysis

Early recurrence analysis was performed in patients with adequate follow-up for 12-month DFS (*n* = 78), excluding those who were censored before 12 months without documented progression. Early recurrence (≤12 months) occurred in a considerable proportion of patients in the eligible cohort. In the univariable logistic regression analysis, preoperative LAR was significantly associated with an increased risk of early recurrence when evaluated as a categorical variable (LAR-high vs. LAR-low: odds ratio [OR] 4.252, 95% CI 1.534–11.783, *p* = 0.005). This association remained robust after adjusting for clinically relevant covariates in the multivariable analysis (OR 4.656, 95% CI 1.520–14.262, *p* = 0.007). In contrast, when LAR was analyzed as a continuous variable, no statistically significant association with early recurrence was observed, suggesting that threshold-based stratification may better capture its clinical relevance than continuous variable analysis. These findings indicate that an elevated preoperative LAR is associated with a markedly increased risk of early recurrence following curative-intent resection (Table 5).

**Figure 4:**
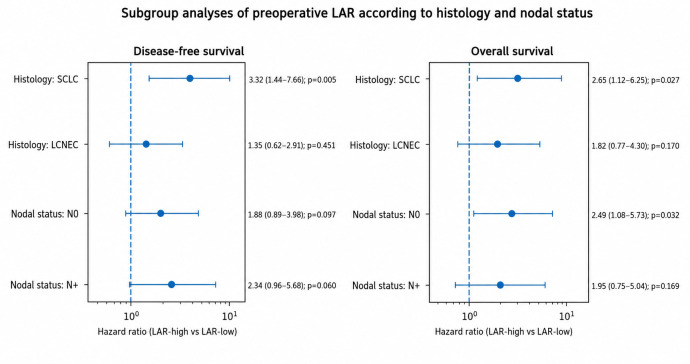
Subgroup Analysis of the Prognostic Impact of Preoperative LAR. Forest plot showing the association between elevated preoperative lactate dehydrogenase-to-albumin ratio (LAR-high vs. LAR-low) and survival outcomes across predefined subgroups. Hazard ratios (HRs) and 95% confidence intervals (CIs) are presented for disease-free survival (DFS) (**left** panel) and overall survival (OS) (**right** panel). Subgroups included histologic subtype (small cell lung cancer [SCLC] vs. large cell neuroendocrine carcinoma [LCNEC]) and nodal status (N0 vs. N+). The vertical dashed line represents the null value (HR = 1). Interaction *p*-values were calculated to assess the potential effect of modification across subgroups.

Subgroup analyses demonstrated that the prognostic effect of LAR was consistent across the predefined subgroups. Although the magnitude of the association appeared more pronounced in patients with SCLC, formal interaction testing did not reveal a statistically significant effect modification by histologic subtype or nodal status. These findings suggest that the prognostic value of LAR is broadly applicable across different clinicopathological subgroups (Figure 4).

## 4. Discussion

In this multicenter cohort of patients undergoing curative-intent resection for pulmonary neuroendocrine carcinomas, preoperative LAR emerged as a clinically informative and biologically integrative biomarker reflecting the interplay between tumor metabolic activity and host systemic conditions. In a disease characterized by marked biological aggressiveness and heterogeneous postoperative outcomes despite similar clinicopathologic staging, LAR demonstrated consistent prognostic relevance across complementary analytical approaches. Notably, it also identified a subgroup of patients at an increased risk of early postoperative failure, suggesting that LAR captures an underlying tumor–host phenotype associated with aggressive disease behavior.

The biological relevance of LAR lies in its ability to integrate key dimensions of tumor metabolism and host physiology into a single, clinically accessible parameter. LDH reflects enhanced glycolytic flux and hypoxia-driven metabolic reprogramming, hallmarks of aggressive tumor biology that have been consistently associated with increased tumor burden and adverse survival outcomes in thoracic malignancies [10,13]. In parallel, serum albumin represents the systemic consequences of cancer-associated inflammation and impaired physiological reserve, with hypoalbuminemia repeatedly linked to worse clinical outcomes across lung cancer populations [16,17]. At a translational level, these associations likely reflect the interplay between tumor-driven metabolic reprogramming and systemic inflammatory signaling, including lactate-mediated immune suppression and cytokine-driven alterations in hepatic protein synthesis. By combining these complementary dimensions, LAR provides a composite measure of tumor-driven metabolic stress and host vulnerability [23]. This integrative characteristic may explain its consistent performance across analytical models compared with conventional inflammation-based indices that capture only a single aspect of the host response. Beyond oncology, LAR has also been investigated as a prognostic biomarker in other clinical settings, including sepsis, where it reflects the interplay between metabolic stress and systemic inflammatory responses, further supporting its biological relevance [29,30].

Although LAR demonstrated only moderate discriminatory performance, this finding should be interpreted within the context of early-stage, surgically treated disease, where outcomes are shaped by multiple interacting biological and treatment-related factors. In such settings, no single biomarker is expected to achieve high discrimination in isolation. Importantly, the value of LAR lies in its reproducibility and stability across endpoints rather than in the magnitude of its discriminatory capacity. This pattern suggests that LAR reflects a fundamental component of disease biology not fully captured by conventional clinicopathologic variables. From a clinical standpoint, biomarkers providing consistent and incremental prognostic information may still offer meaningful value when incorporated into multivariable risk assessment strategies.

A key finding of this study was the independent association between elevated preoperative LAR and early recurrence. Patients with a high LAR experienced a markedly increased risk of relapse within the first 12 months, even after adjusting for established prognostic factors. Early recurrence represents a clinically distinct and high-risk trajectory, often reflecting aggressive tumor biology and the presence of occult micrometastatic disease at the time of surgery. The association between elevated LAR and early relapse supports the concept that metabolic activation and systemic inflammatory dysregulation contribute to disease progression. In this context, LAR may serve as a surrogate marker of a pre-existing tumor–host state that predisposes patients to rapid postoperative failure. Clinically, this finding suggests that the LAR may help refine postoperative surveillance strategies and identify patients who may benefit from closer follow-up or consideration of adjuvant treatment approaches. In addition to generalized micrometastatic spread, early recurrence may also reflect the presence of underrecognized metastatic sites at the time of diagnosis. Cardiac involvement, although uncommon, represents a potentially overlooked site of metastasis in thoracic malignancies. In this context, echocardiographic evaluation may serve as a first-line, noninvasive tool to identify suspicious findings requiring further imaging in selected patients [31].

Our findings align with the accumulating evidence supporting the prognostic role of composite biomarkers integrating inflammation, nutritional status, and tumor metabolism across malignancies [19,21,23]. However, data on resectable pulmonary neuroendocrine carcinomas remain limited [18,22]. By demonstrating consistent performance in this setting, the present study extends the existing biomarker frameworks to a clinically distinct population. In contrast to conventional indices such as NLR [18], SII [21], and SIRI [25], which reflect isolated inflammatory pathways, LAR integrates both tumor-derived and host-related processes. This distinction may underlie its more stable performance and reinforce its relevance in tumors characterized by metabolic reprogramming and systemic inflammatory activation.

From a clinical perspective, LAR is simple, cost-effective, and readily available, allowing for seamless integration into routine preoperative assessments. Its ability to stratify patients according to the recurrence risk, particularly early recurrence, may support more individualized postoperative management. Patients with an elevated LAR may benefit from closer surveillance, especially during the first postoperative year, and could be considered for intensified or tailored adjuvant strategies pending prospective validation. In addition, LAR may have potential utility in clinical trial design, particularly for risk stratification and enrichment of high-risk populations in perioperative settings.

This study has several limitations. The retrospective design introduces the possibility of selection bias and residual confounding, although the multicenter structure may mitigate some of these concerns. In addition, potential factors influencing LDH levels, including certain medications and comorbid conditions, could not be fully accounted for due to the retrospective design, representing a potential source of residual confounding. Variability in clinical practice across centers may also have influenced the outcomes. In addition, no formal a priori sample size calculation was performed due to the retrospective design, and all consecutive eligible patients were included, which may limit statistical power. The relatively limited sample size, particularly within subgroup analyses, further reduces the ability to detect interaction effects and warrants cautious interpretation of the findings. Although internal validation using bootstrap resampling supported model stability, the use of a cutoff derived from the same cohort and the relatively limited sample size may still introduce optimism bias. Therefore, the findings should be interpreted with caution and require external validation in independent populations.

## 5. Conclusions

In conclusion, this multicenter real-world study suggests that preoperative LAR is a biologically grounded and clinically informative biomarker associated with survival and early recurrence in resected pulmonary neuroendocrine carcinomas. By capturing the interaction between tumor metabolic activity and host systemic conditions, LAR may provide complementary prognostic information in settings characterized by substantial outcome heterogeneity. However, its discriminatory performance remains moderate, and the findings should be interpreted with caution, given the limited sample size and the use of a cohort-derived cutoff. Prospective validation in larger independent cohorts is required before its integration into routine clinical decision-making.

## Figures and Tables

**Table 2:** Receiver Operating Characteristic Performance and DeLong Comparisons of Systemic Inflammatory Biomarkers for 12-month DFS and 24-month OS.

Marker	DFS AUC (95% CI)	Cut-Off	Sensitivity	Specificity	OS AUC (95% CI)	Sensitivity	Specificity	DeLong p vs. LAR
LAR	0.603 (0.468–0.729)	45.581	0.782	0.543	0.679 (0.544–0.800)	0.762	0.543	—
CAR	0.574 (0.448–0.704)	1.0	0.752	0.435	0.609 (0.487–0.749)	0.745	0.486	0.363
GINI	0.571 (0.448–0.71)	410.408	0.782	0.478	0.616 (0.485–0.759)	0.792	0.543	0.442
MLR	0.535 (0.392–0.678)	0.455	0.283	0.935	0.510 (0.366–0.642)	0.174	0.857	0.050
PIV	0.520 (0.392–0.656)	890.567	0.188	0.935	0.571 (0.436–0.706)	0.151	0.914	0.261
SII	0.516 (0.383–0.65)	1331.25	0.156	0.957	0.536 (0.407–0.667)	0.155	0.971	0.130
NLR	0.501 (0.357–0.629)	3.577	0.283	0.891	0.500 (0.357–0.629)	0.228	0.857	0.041
SIRI	0.493 (0.345–0.637)	2.481	0.346	0.826	0.518 (0.375–0.651)	0.226	0.714	0.076

**Abbreviations:** AUC, area under the curve; DFS, disease-free survival; OS, overall survival. Optimal cut-off values were derived from the 12-month DFS ROC curves using the Youden index and subsequently applied to both DFS and OS analyses. DeLong tests were used to compare the areas under the ROC curves of each biomarker against LAR.

**Table 3:** Univariate Cox Regression Analysis of DFS and OS in Resected Pulmonary Neuroendocrine Carcinomas.

Variable	Disease-Free Survival		Overall Survival	
	HR (95% CI)	*p*	HR (95% CI)	*p*
Age	1.007 (0.975–1.041)	0.658	1.031 (0.996–1.067)	0.081
Sex (female vs. male)	0.897 (0.437–1.840)	0.767	0.863 (0.386–1.927)	0.719
ECOG (≥2 vs. 0–1)	1.632 (0.502–5.305)	0.415	3.08 (0.908–10.443)	0.071
Smoking (yes vs. no)	1.693 (0.653–4.395)	0.279	1.651 (0.570–4.782)	0.355
Comorbidity (yes vs. no)	1.777 (0.938–3.366)	0.078	2.054 (1.025–4.117)	0.042
Tumor laterality (right vs. left)	0.943 (0.561–1.588)	0.826	0.826 (0.474–1.440)	0.500
Histology (LCNEC vs. SCLC)	0.618 (0.366–1.044)	0.072	0.695 (0.398–1.214)	0.201
Preoperative radiologic mediastinal LN positivity (positive vs. negative)	1.608 (0.952–2.716)	0.076	1.498 (0.853–2.633)	0.160
Type of resection (pneumonectomy vs. segmentectomy/lobectomy)	1.219 (0.485–3.064)	0.674	1.152 (0.413–3.212)	0.787
Type of surgery (open vs. VATS)	1.0 (0.545–1.833)	1.000	1.299 (0.649–2.603)	0.460
pT subgroup (T3–4 vs. T1–2)	0.903 (0.442–1.844)	0.779	1.08 (0.524–2.228)	0.834
pN stage (N+ vs. N0)	1.954 (1.149–3.322)	0.013	1.63 (0.923–2.876)	0.092
Lymphovascular invasion	1.421 (0.833–2.426)	0.197	1.465 (0.827–2.595)	0.191
Perineural invasion	1.534 (0.859–2.742)	0.148	1.622 (0.882–2.984)	0.120
Clinical stage	1.543 (0.782–3.043)	0.211	1.746 (0.866–3.520)	0.119
Primary tumor diameter	0.997 (0.985–1.010)	0.672	1.003 (0.990–1.016)	0.622
Brain metastasis at progression (yes vs. no)	1.137 (0.655–1.972)	0.649	1.489 (0.832–2.666)	0.180
Adjuvant chemotherapy (yes vs. no)	0.665 (0.344–1.285)	0.225	0.632 (0.314–1.270)	0.198
Adjuvant radiotherapy (yes vs. no)	1.278 (0.740–2.207)	0.378	1.14 (0.634–2.050)	0.661
LAR	1.012 (1.002–1.023)	0.024	1.017 (1.007–1.027)	0.001
NLR	0.98 (0.829–1.158)	0.811	1.01 (0.861–1.184)	0.905
SII	1.0 (0.999–1.001)	0.934	1.0 (0.999–1.001)	0.714
SIRI	1.14 (0.885–1.469)	0.311	1.244 (0.968–1.599)	0.088
PIV	1.0 (0.999–1.001)	0.816	1.0 (1.000–1.001)	0.357
CAR	0.992 (0.957–1.029)	0.675	0.996 (0.961–1.033)	0.840
GINI	1.0 (1.000–1.000)	0.529	1.0 (1.000–1.000)	0.722

**Abbreviations**: CAR, C-reactive protein-to-albumin ratio; CI, confidence interval; ECOG, Eastern Cooperative Oncology Group; GINI, GINI index; HR, hazard ratio; LAR, lactate dehydrogenase-to-albumin ratio; LCNEC, large cell neuroendocrine carcinoma; NLR, neutrophil-to-lymphocyte ratio; OS, overall survival; pN, pathologic nodal stage; pT, pathologic tumor stage; PIV, pan-immune-inflammation value; SCLC, small cell lung cancer; SII, systemic immune-inflammation index; SIRI, systemic inflammation response index; VATS, video-assisted thoracoscopic surgery.

**Table 4:** Multivariable Cox Regression Model of DFS and OS in Resected Pulmonary Neuroendocrine Carcinomas.

Variable	Disease-Free Survival				Overall Survival			
	Model 1 HR (95% CI)	*p*	Model 2 HR (95% CI)	*p*	Model 1 HR (95% CI)	*p*	Model 2 HR (95% CI)	*p*
Age	0.999 (0.965–1.035)	0.953	0.996 (0.961–1.032)	0.823	1.022 (0.984–1.061)	0.266	1.018 (0.978–1.058)	0.384
ECOG (≥2 vs. 0–1)	1.963 (0.559–6.891)	0.292	1.922 (0.546–6.765)	0.309	4.031 (1.096–14.821)	0.036	3.958 (1.074–14.584)	0.039
Comorbidity (yes vs. no)	1.693 (0.866–3.309)	0.124	1.641 (0.834–3.228)	0.152	2.098 (1.026–4.291)	0.042	1.968 (0.950–4.075)	0.068
Histology (LCNEC vs. SCLC)	0.665 (0.367–1.208)	0.181	0.629 (0.341–1.161)	0.138	0.674 (0.347–1.308)	0.243	0.62 (0.309–1.242)	0.177
pT stage subgroup (T1–2 vs. T3–4)	1.216 (0.549–2.690)	0.63	1.253 (0.565–2.779)	0.579	1.55 (0.682–3.524)	0.296	1.634 (0.715–3.733)	0.245
pN stage (N0 vs. N+)	1.565 (0.894–2.741)	0.117	1.552 (0.878–2.743)	0.130	1.336 (0.711–2.510)	0.368	1.323 (0.692–2.529)	0.397
LAR	1.012 (1.001–1.023)	0.037	1.011 (1.000–1.022)	0.044	1.016 (1.005–1.027)	0.003	1.015 (1.005–1.026)	0.005
SIRI	-	-	1.162 (0.888–1.521)	0.272	-	-	1.199 (0.900–1.597)	0.215

**Abbreviations**: CI, confidence interval; ECOG, Eastern Cooperative Oncology Group; SCLC, small cell lung cancer; LCNEC, large cell neuroendocrine carcinoma; HR, hazard ratio; LAR, lactate dehydrogenase-to-albumin ratio; SIRI, systemic inflammation response index; pT, pathological tumor stage; pN, pathological nodal stage. Hazard ratios (HRs) and 95% confidence intervals (CIs) were estimated using Cox proportional hazards regression models. Statistical significance was set at *p* < 0.05. Model 1 was adjusted for age, ECOG performance status, comorbidities, histology, pathologic T stage, and pathologic nodal status. Model 2 additionally included the SIRI.

**Table 5:** Association Between Preoperative LAR and Early Recurrence.

Variable	Model	OR (95% CI)	*p*
LAR (high vs. low)	Univariable	4.252 (1.534–11.783)	0.005
LAR (high vs. low)	Multivariable Model 1	4.656 (1.520–14.262)	0.007
LAR (per 10-unit increase)	Univariable	1.160 (0.938–1.435)	0.170
LAR (per 10-unit increase)	Multivariable Model 1	1.172 (0.937–1.464)	0.164

**Abbreviations**: CI, confidence interval; LAR, lactate dehydrogenase-to-albumin ratio; OR, odds ratio. Early recurrence was defined as disease recurrence within 12 months of surgery. Patients who were censored before 12 months without documented progression were excluded. Multivariable Model 1 was adjusted for age, ECOG performance status, comorbidity, histologic subtype, pathologic T stage, and pathologic nodal status.

## Data Availability

The datasets generated and/or analyzed during the present study are available from the corresponding author upon reasonable request, contingent upon approval by the Department of Thoracic Surgery at the University of Health Sciences Antalya Training and Research Hospital.

## Abbreviations

The following abbreviations are used in this manuscript:

AUC	Area under the curve
CAR	C-reactive protein-to-albumin ratio
CRP	C-reactive protein
DFS	Disease-free survival
ECOG	Eastern Cooperative Oncology Group
GINI	Global immune-nutrition-inflammation index
IPCW	Inverse probability of censoring weighting
IQR	Interquartile range
LAR	Lactate dehydrogenase-to-albumin ratio
LCNEC	Large cell neuroendocrine carcinoma
LDH	Lactate dehydrogenase
MLR	Monocyte-to-lymphocyte ratio
NEC	Neuroendocrine carcinoma
NLR	Neutrophil-to-lymphocyte ratio
OS	Overall survival
PIV	Pan-immune-inflammation value
PNI	Prognostic nutritional index
ROC	Receiver operating characteristic
SCLC	Small cell lung cancer
SII	Systemic immune-inflammation index
SIRI	Systemic inflammation response index
